# TRIM25 Suppresses Rabies Virus Fixed HEP-Flury Strain Production by Activating RIG-1-Mediated Type I Interferons

**DOI:** 10.3390/genes14081555

**Published:** 2023-07-29

**Authors:** Boyue Zhang, Ting Cai, Hongling He, Xuezhe Huang, Yongwen Luo, Shile Huang, Jun Luo, Xiaofeng Guo

**Affiliations:** 1 College of Veterinary Medicine, South China Agricultural University, Guangzhou 510651, China; zhangboyue163@163.com (B.Z.); 18819773609@163.com (T.C.); 13610205214@163.com (H.H.); scauhxz@163.com (X.H.); ywluo@scau.edu.cn (Y.L.); junluo@scau.edu.cn (J.L.); 2 Department of Biochemistry and Molecular Biology, Louisiana State University Health Sciences Center, 1501 Kings Highway, Shreveport, LA 71130-3932, USA; shuan1@lsuhsc.edu; 3 Feist-Weiller Cancer Center, Louisiana State University Health Sciences Center, Shreveport, LA 71130-3932, USA

**Keywords:** RIG-I, rabies virus, TRIM25, interferon, ubiquitination

## Abstract

Rabies remains a great threat to public health worldwide. So far, the mechanism of rabies virus (RABV) infection is not fully understood, and there is no effective treatment for rabies. Identifying more host restriction factors of RABV will spur the development of novel therapeutic interventions against rabies. Accumulating studies suggest that tripartite motif-containing (TRIM) proteins have great effects on virus replication. TRIMs control the antiviral responses through either direct interaction with viral proteins or indirect regulation of innate immune signaling molecules in the host. The role of TRIM25 in rabies virus (RABV) infection is poorly understood. Using next-generation sequencing, we found that TRIM25 is upregulated during HEP-Flury infection. Knockdown of TRIM25 enhances HEP-Flury production, while overexpression of TRIM25 suppresses HEP-Flury replication. Knockdown of interferon α and interferon β weakens the anti-RABV response induced by TRIM25 overexpression, and potentiates RABV production. Furthermore, we found that TRIM25 regulates type-I interferon response by targeting retinoic acid-inducible gene I (RIG-I) during HEP-Flury infection. Knockdown of RIG-I weakens the anti-HEP-Flury response induced by TRIM25 overexpression, indicating that TRIM25 regulates RABV production via the RIG-I-IFN axis. In addition, we observed that TRIM25 does not directly interact with HEP-Flury structural proteins, suggesting that TRIM25 regulates HEP-Flury production indirectly. Taken together, our work identifies TRIM25 as a new host factor involved in HEP-Flury infection, which may be a potential target for the development of antiviral drugs against RABV.

## 1. Introduction

Rabies is an acute inflammation of the nervous system caused by Rabies virus (RABV) infection. The fatality rate is more than 99.9% once clinical symptoms appear. Worldwide, it is estimated that 59,000 people die from rabies annually [1]. The RABV genome sequence is approximately 12 kb, including encoding nucleoprotein (N), phosphoprotein (P), matrix protein (M), glycoprotein (G) and RNA-dependent RNA polymerase (L). The all-structural protein of RABV is the key of the virus’ ability to produce protein [2]. Although many studies have focused on the mechanism of RABV infection and the interaction between RABV and host factors, current knowledge regarding the therapeutic targets of rabies is limited. Thus, it is essential to find more host anti-RABV factors in order to clarify the underlying mechanisms of viral infection, providing a new strategy for the treatment of RABV.

The host’s inherent immune responses are the primary defense against infections. Notably, the type I interferon (IFNα/β) is an important innate immune response factor for inhibiting RABV proliferation [3,4,5,6]. Furthermore, wild-type RABV fails to induce type I interferon response, which further demonstrates the important role of IFNα/β in the control of RABV infection [7]. Evasion of the innate immune response by RABV is conducted by viral proteins including N, P and M [8,9,10]. In addition, critical mutations in G and L regulate the induction of type-I IFN [11,12]. It has been shown that IFNα/β potentiates the adaptive immune response in RABV infection and subsequently suppresses viral production [13,14,15]. Invasive RABV is recognized by intracellular pattern recognition receptors (PRRs), including Toll-like receptors, melanoma differentiation-associated protein 5 (MDA5), and retinoic acid-inducible gene I (RIG-I), which subsequently activate interferon regulatory factors (IRFs) [16,17].

Ubiquitination is one of the forms of post-translational modification, which is important in the localization, metabolism, function and degradation of proteins. Ubiquitination modification is involved in the regulation of almost all life-essential cellular activities, such as cell proliferation, apoptosis, autophagy, gene expression, signal transduction, immune response, inflammatory response, and protein degradation/non-degradation modification [18]. E3 ubiquitin ligase plays a great part in the process of ubiquitination [19]. Notably, tripartite motif (TRIM) proteins are a large group of E3 ubiquitin ligases involved in the regulation of virus replication [20]. Antiviral mechanisms for TRIM proteins include directly targeting viral components or regulating innate immune responses [21,22]. The modification of type-I IFN pathway proteins by TRIM proteins is extensive, including almost all the upstream and downstream pathway proteins of type-I IFN, which may subsequently suppress or promote virus replication [23,24,25]. For example, TRIM13 is a specific negative regulator of MDA5-mediated type-I IFN production during encephalomyocarditis virus infection [26]. TRIM4 positively regulates RIG-I mediated type-I IFN response during virus infection [27]. However, some TRIM proteins display a double-sided effect on type-I IFN regulation. For instance, one study showed that TRIM21 enhanced IRF3-mediated antiviral response [28], while another study showed that TRIM21 inhibited IRF3-dependent type-I IFN production by targeting the transcription factor for polyubiquitination [29]. TRIM25 is the first identified immunomodulatory factor of the TRIM protein family. On the one hand, TRIM25 positively regulates the innate immune response by targeting RIG-I and MDA5 during virus infection [30,31]. On the other hand, TRIM25 inhibits virus replication by directly targeting viral proteins [30,31,32]. However, little is known regarding whether TRIM 25 regulates RABV replication. In general, TRIM proteins have different mechanisms in regulating virus-induced type-I IFN in response to the infections of different viruses [33].

In order to identify more mechanisms regulating host infection by RABV, we performed transcriptome analysis of RABV-infected mouse neuroblastoma (NA) cells. We found that the expression of some Trim proteins was altered, especially Trim25, which was significantly changed after HEP-Flury infection. The focus of this study was to investigate the function of TRIM25 in HEP-Flury infection. Our findings suggest that TRIM25 inhibits HEP-Flury infection by activating the RIG-I-mediated type-I IFN response.

## 2. Results

### 2.1. The Infection with HEP-Flury Leads to Increased Expression of TRIM25 in N2a Cells

**Figure 1:**
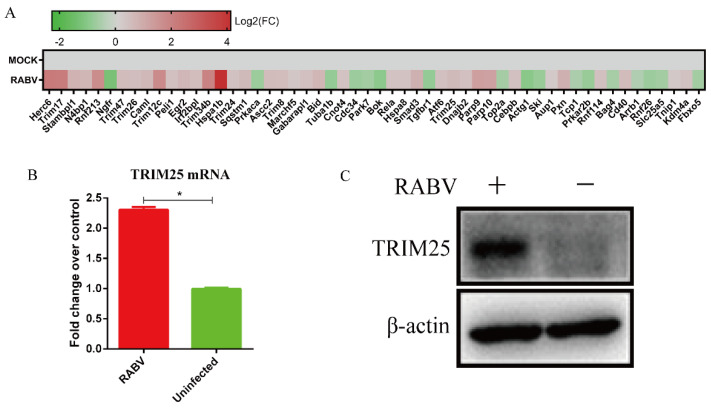
The expression of TRIM25 was upregulated after HEP-Flury infection. (**A**) RNA−seq analysis of differential expression of ubiquitination-related host molecules in N2a cells 24 h after HEP-Flury infection compared with uninfected cells (*p* < 0.01). HEP-Flury was inoculated at an MOI of 0.5 in the NA2 cells of the infection group. After 24 h of infection, total RNA was isolated from infected and uninfected cells, and then analyzed by qRT-PCR to determine the transcription level of TRIM25 mRNA. (**B**). Detection of TRIM25 protein levels in whole cell lysates by Western blot using an anti-TRIM25 antibody (**C**). Data are presented as mean ± SD; *, *p* < 0.05.

To systematically characterize HEP-Flury potential host regulators, we analyzed differentially expressed genes based on RNA-seq data of HEP-Flury-infected N2a cells (NCBI Sequence Read Archive database accession number: PRJNA898812). The results showed that several ubiquitin-associated proteins, particularly some E3 ubiquitin ligases, were significantly upregulated or downregulated upon RABV infection compared with non-infection (*p* < 0.01) (Figure 1A). In order to confirm the finding, N2a cells were infected with 0.5 MOI of HEP-Flury for 24 h, followed by RT-qPCR and Western blotting. We found that HEP-Flury infection significantly increased the mRNA (Figure 1B) and protein levels of TRIM25 (Figure 1C). The results suggest that HEP-Flury infection increases the expression of TRIM25.

### 2.2. Trim25 Negatively Regulates HEP-Flury Replication

**Figure 2:**
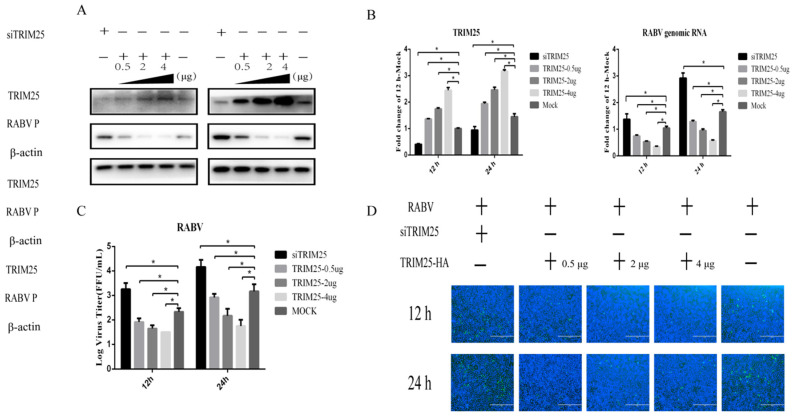
TRIM25 negatively regulates the replication of HEP-Flury. N2a cells were transfected with TRIM25 siRNA or three different concentrations of pCAGGS-HA-TRIM25 plasmid (0.5 μg, 2 μg or 4 μg) for 24 h, and then the cells were infected with HEP-Flury at an MOI of 0.5 for 12 h or 24 h. Non-targeting control siRNA (NC) or empty vector plasmid (pCAGGS-HA) was used as the experimental control group. (**A**) Treated cell lysates were analyzed by Western blot using antibodies against TRIM25, HEP-Flury P, and β-actin. (**B**) Total RNA in cells was collected and the transcript levels of HEP-Flury genomic RNA and TRIM25 mRNA were detected by RT-qPCR. (**C**) Supernatants from infected cells were collected and virus titers were determined. (**D**) Cells infected with HEP-Flury were fixed and incubated with FITC-labeled anti-RABV-N antibody (green) and DAPI (blue), followed by immunofluorescence to detect RABV N protein. Results are expressed as mean ± SD; *, *p* < 0.05.

TRIM25 is widely involved in the host’s resistance to viral infection [34,35,36]. Based on the observed expression level of TRIM25 after HEP-Flury infection, we speculated that TRIM25 may be a key factor in host response to HEP-Flury infection. In order to verify this speculation, we used the overexpression of TRIM25 and TRIM25 small interfering RNA (siTRIM25). Knockdown of Trim25 increased HEP-Flury P protein levels (Figure 2A), RNA genome levels (Figure 2B), virus titers (Figure 2C) and N protein levels detected by immunofluorescence at 12 and 24 h after HEP-Flury infection. In contrast, TRIM25 overexpression reduced HEP-Flury P protein levels, RNA genome levels, viral titers, and immunofluorescence detection levels of N protein at 12 and 24 h after HEP-Flury infection (Figure 2). TRIM25 resists the replication of HEP-Flury in a dose-dependent manner. The results indicated that TRIM25 negatively regulates the replication of HEP-Flury.

### 2.3. TRIM25 Suppresses HEP-Flury Reproduction by Promoting Type I Interferon Expression

**Figure 3:**
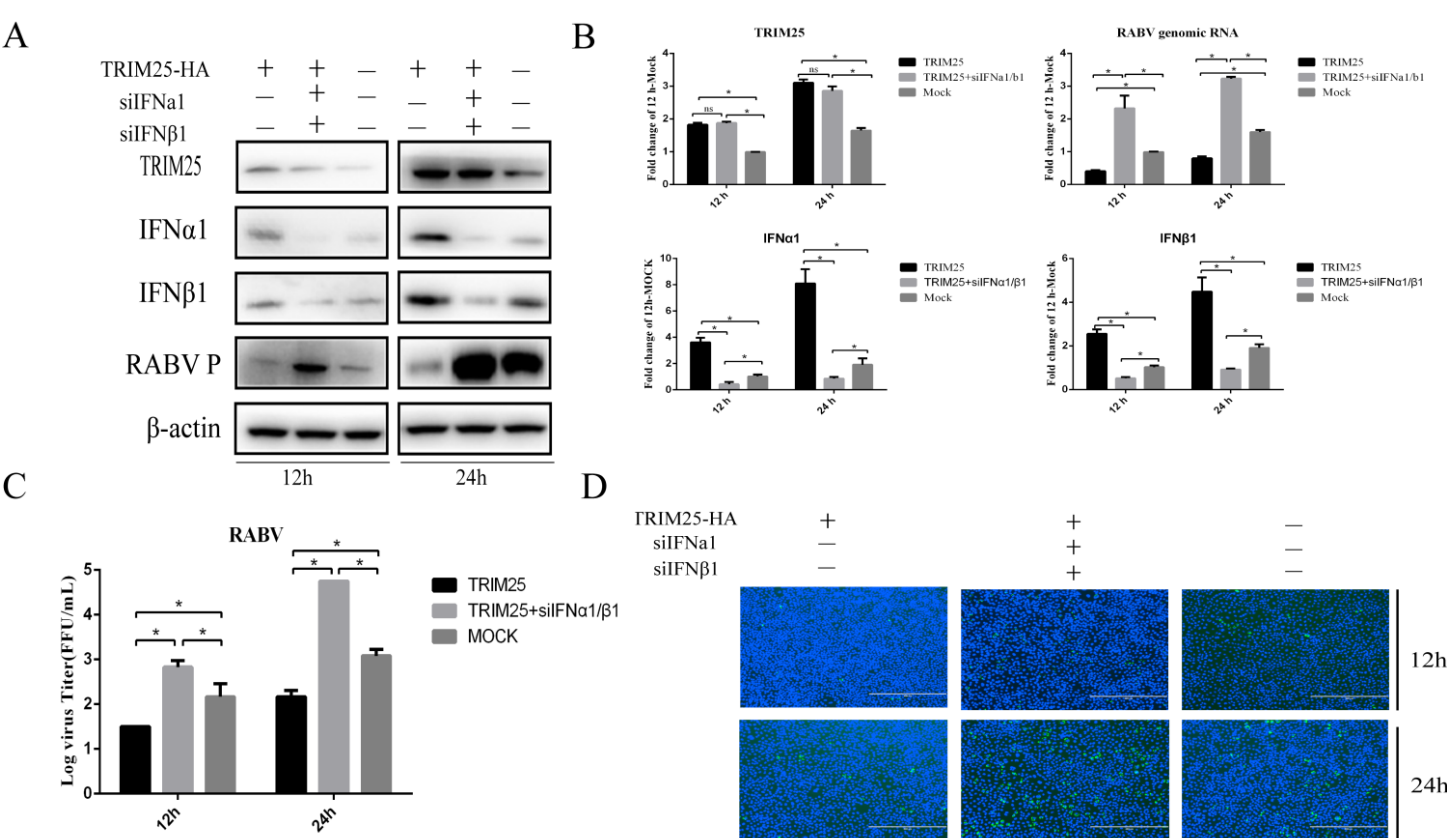
TRIM25 inhibits the replication of HEP-Flury by promoting the expression of type-I IFN. N2a cells were transfected with small siRNA (1 μg) (siIFN α and siIFN β) or pCAGGS-HA-TRIM25 plasmid (1 μg) for 24 h, and then infected with HEP-Flury at an MOI of 0.5 for 12 h and 24 h. (**A**) Protein levels of TRIM25, HEP-Flury P, IFNα and IFNβ in whole cell lysates were analyzed by Western blotting. (**B**) Total RNA of treated cells was collected, and the mRNA levels of TRIM25, HEP-Flury genomic RNA, IFNα, and IFNβ were detected by RT-qPCR. (**C**) TCID50 assay was used to detect the virus titers released from cells with different treatments. (**D**) Fixed cells were stained with FITC-labeled anti-RABV-N antibody (green) and DAPI (blue), followed by immunofluorescence detection of HEP-Flury N protein. Results are expressed as mean ± SD; *, *p* < 0.05; ns, not significant.

Type I interferons play a key role in clearing RABV during viral infection [16,17]. Small siRNAs (siIFN-α and siIFN-β) were used to determine whether TRIM25 inhibits HEP-Flury propagation through the IFN signaling pathway. We found that overexpression of TRIM25 significantly reduced the level of HEP-Flury replication at 12 h and 24 h post infection (hpi) (Figure 3). Interestingly, knockdown of IFNα and IFNβ using small siRNAs (siIFNα and siIFNβ) significantly reduced the inhibitory effects of TRIM25 overexpression on HEP-Flury P protein levels (Figure 3A), genomic RNA (Figure 3B), viral titers (Figure 3B,C), and HEP-Flury N protein immunofluorescence at 12 and 24hpi (Figure 3D). The experimental results indicated that TRIM25 suppressed HEP-Flury reproduction by increasing the expression of type-I IFN.

### 2.4. TRIM25 Regulates the Expression of IFN through RIG-I after HEP-Flury Infection

**Figure 4:**
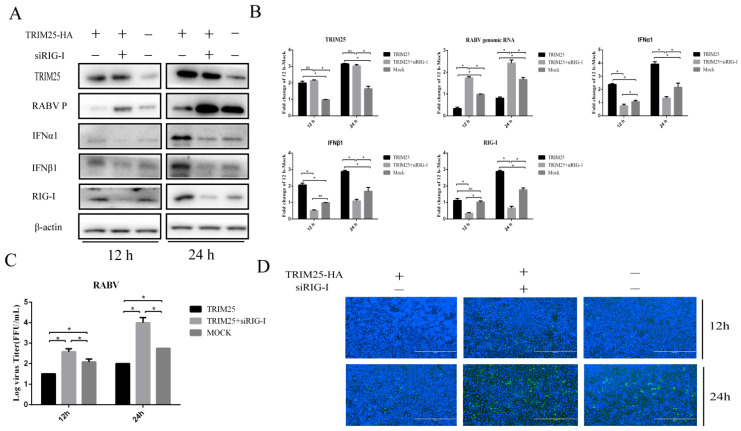
TRIM25 regulates the expression of IFN through RIG-I after HEP-Flury infection. N2a cells were transfected with small siRNA (1 μg) (siRIG-I) or pCAGGS-HA-TRIM25 plasmid (1 μg) for 24 h, and then infected with HEP-Flury at an MOI of 0.5 for 12 h and 24 h. The control group was treated in parallel with pCAGGS-HA or NC. (**A**) Protein levels of TRIM25, HEP-Flury P, IFNα, IFNβ and RIG-I in whole cell lysates were analyzed by Western blotting. (**B**) Total RNA of treated cells was collected, and the mRNA levels of TRIM25, HEP-Flury genomic RNA, IFNα, IFNβ and RIG-I were detected by RT-qPCR. (**C**) TCID50 assay was used to detect the virus titers released from cells with different treatments. (**D**) Fixed cells were stained with FITC-labeled anti-RABV-N antibody (green) and DAPI (blue), followed by immunofluorescence detection of HEP-Flury N protein. Results are expressed as mean ± SD; *, *p* < 0.05; ns, not significant.

It is well known that RIG-I plays a key role in the induction of metaphase interferon production during HEP-Flury infection [10]. We wanted to verify whether TRIM25-mediated expression of type-I IFN in HEP-Flury infection was dependent on RIG-I. To this end, RIG-I was knocked down (siRIG-I) while overexpressing TRIM25. Then, the expression of IFN-α/β was measured 12 or 24 h after HEP-Flury infection of N2a cells. Consistent with the above results, the protein levels of IFNα and IFNβ were upregulated after overexpression of TRIM25 (Figure 4A,B). Moreover, knockdown of RIG-I attenuated TRIM25-induced IFNα and IFNβ expression (Figure 4A,B). These data suggest that TRIM25 upregulates type-I IFN expression through RIG-I during HEP-Flury infection. Next, we confirmed whether TRIM25 truly regulates the replication of HEP-Flury through RIG-I. The results showed that knockdown of RIG-I significantly reduced TRIM25-mediated suppression trend of HEP-Flury P protein level (Figure 4A), genomic RNA level (Figure 4B), virus titer (Figure 4C) and N protein immunofluorescence fluorescence level of RABV. (Figure 4D). In general, our experimental results demonstrate that TRIM25 regulates HEP-Flury replication through the RIG-I-IFN axis.

### 2.5. HEP-Flury Does Not Directly Interact with TRIM25

**Figure 5:**
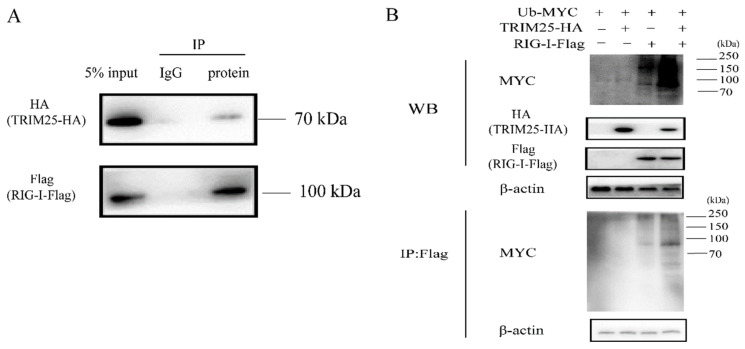
TRIM25 ubiquitinates RIG-I through direct interaction. (**A**) RIG-I directly interacts with TRIM25. Flag-RIG-I and HA-TRIM25 plasmids were co-transfected into HEK 293T cells, and the cells were collected 36 h later. The cell lysates were incubated with conjugated magnetic beads loaded with normal IgG or anti-FLAG antibody at 4 °C for 10 h. The collected cell lysates and IP products were subjected to Western blot analysis with anti-Flag antibody and anti-HA antibody. (**B**) TRIM25 ubiquitinates RIG-I. HEK 293T cells were transfected with Flag-RIG-I, Myc-Ub, and HA-TRIM25 plasmids for 36 h. Western blots of whole-cell lysates or Flag immunoprecipitates were analyzed using specific antibodies.

**Figure 6:**
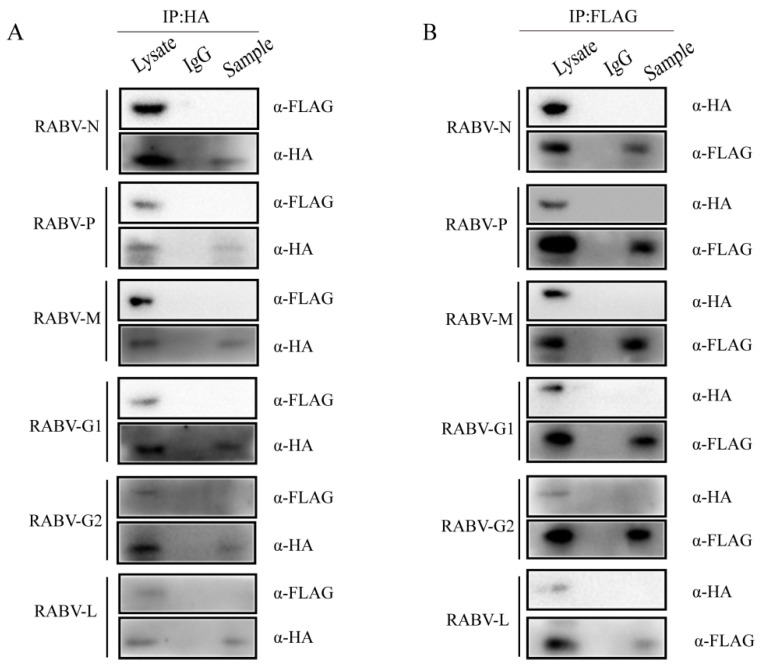
TRIM25 does not directly interact with the structural proteins of HEP-Flury. (**A**,**B**) HEK 293T cells were transfected with Flag-N, P, M, G or L plasmids and HA-TRIM25. (**A**) Cell lysates were incubated with anti-HA antibody or normal IgG-conjugated magnetic beads at 4 °C 10 h. The IP products were detected by Western blot with anti-HA antibody and anti-Flag antibody. (**B**) Normal IgG or anti-FLAG antibody-conjugated magnetic beads and cell lysates were incubated at 4 °C for 10 h. IP products were detected by Western blot with anti-HA antibody and anti-Flag antibody.

Recent studies have shown that TRIM25 plays a role in the ubiquitination of RIG-I [37,38]. To further investigate the role of the mechanism of TRIM25 regulating the RIG-I-IFN axis during HEP-Flury infection, we first confirmed that TRIM25 mediates RIG-I ubiquitination. As shown in Figure 5A, RIG-I interacts with TRIM25. Overexpression of TRIM25 can significantly enhance the ubiquitination level of RIG-I (Figure 5B), which is consistent with previous findings [39,40]. Since TRIM25 expression can be targeted by viral proteins through different mechanisms [41,42,43], in order to further investigate whether HEP-Flury structural proteins target TRIM25 and whether TRIM25 directly targets viral proteins for ubiquitination to limit HEP-Flury replication, HA-TRIM25 was co-expressed with HEP-Flury viral protein in 293T cells. All these results confirmed that the Flag-tagged structural protein of HEP-Flury was not detected in the immunoprecipitant with HA antibody (Figure 6A), while HA-TRIM25 was not detected in the immunoprecipitant with Flag antibody (Figure 6B). These results indicate that none of the HEP-Flury structural proteins (N, P, M, G, and L) physically interact with TRIM25.

## 3. Discussion

RABV infection causes almost 100% mortality, with no treatment being available thus far. RABV infection causes almost 100% mortality when no treatment is available. Therefore, it is very important to explore the host factors that regulate RABV infection. In the present study, we observed a strong upregulation of TRIM25 expression after HEP-Flury infection. Moreover, TRIM25 is a limiting factor for HEP-Flury infection. In fact, many members of the TRIM protein family exhibit certain antiviral effects. TRIM21 is upregulated both in vivo and in vitro during viral infection, significantly inhiting porcine reproductive and respiratory syndrome virus (PRRSV) replication [44]. Infection with influenza A virus (IAV) and hepatitis C virus induces upregulation of TRIM22, which in turn limits viral replication [45,46]. White spot syndrome virus infection upregulates the expression of TRIM32, which conversely shows antiviral activity [47]. Infectious bursal disease virus (IBDV) infection induces a marked upregulation of TRIM25 expression, thereby limiting viral replication [32]. Here, we found that RABV also upregulates TRIM25, which conversely inhibits the viral replication. This is likely a negative feedback mechanism. A study has confirmed that TRIM62 inhibits the replication of avian leukosis virus, which firstly upregulates TRIM62 in the early stage of infection and then downregulates TRIM62 during the late stage of infection [48]. In our current study, exogenous overexpression of TRIM25 produced a significant inhibitory effect on the replication of HEP-Flury. Our results demonstrate that TRIM25 is a novel host factor that restricts HEP-Flury replication and warrants further investigation.

The level of a protein can be regulated at transcriptional, translational, and post-translational levels [49]. This study found that infection with HEP-Flury upregulated the transcription level of TRIM25 mRNA nearly 2-fold, and the protein level of TRIM25 was significantly increased according to Western blot detection (Figure 1). It is possible that RABV infection increases the protein level of TRIM25 not only by increasing its mRNA expression, but also by increasing its protein synthesis and/or by decreasing its protein degradation. Undoubtedly, further research is needed to address this question.

The mechanism of antiviral activity caused by host factors is associated with either the restriction of viral replication, including binding, entry, synthesis, assembly and budding, or innate immune response. TRIM25 regulates innate immune response through several mechanisms in viral infection [22]. Our study found that overexpression of TRIM25 during HEP-Flury infection induced upregulation of IFNα and IFNβ expression levels, thereby limiting viral replication. Knockdown of IFNα and IFNβ significantly increased HEP-Flury production in N2a cells, providing evidence in support of this notion, and consistent with another study [4].

Type-I IFN production is regulated by TRIM25 through either RIG-I or MDA5 [30,40,42,50]. Our study indicated that TRIM25 positively regulates type-I IFN in a RIG-I-dependent manner in response to RABV infection. Therefore, TRIM25 mediates anti-RABV activity through the RIG-I-IFN axis. This study reveals the mechanism of type-I-IFN-mediated anti-RABV activity. Whether other pattern recognition receptors such as MDA5 act as targets for TRIM25 in RABV infection will be investigated in future study.

Previous studies have indicated that TRIM25 delivers polyubiquitin chains to RIG-I, which induces RIG-I-mediated antiviral activity [38]. In this study, we confirmed that TRIM25 is involved in the ubiquitination of RIG-I through a direct interaction, which regulates type-I-IFN-mediated antiviral response. Besides modulating cellular signaling cascades of the antiviral innate immune response, TRIM proteins also directly target viral components [21]. For example, TRIM11 interacts with HIV-1 capsid and subsequently accelerates HIV-1 uncoating and reduces viral reverse transcription [51]. TRIM21 inhibits hepatitis virus (HBV) replication by promoting ubiquitination of HBV DNA polymerase [52]. TRIM14, TRIM22 and TRIM41 have been demonstrated to inhibit IAV by targeting viral nucleoprotein for degradation [46,53,54]. Notably, a previous study showed that TRIM25 can directly target the structural protein VP3 while simultaneously degrading the protein through ubiquitination, thereby inhibiting the replication of IBDV [32]. Moreover, a recent study has also shown that TRIM25 directly interacts with the P protein of the CVS-B2c strain of RABV [55]. In the present study, we found that RABV proteins of the HEP-Flury strain did not directly target TRIM25. The biological characteristics of different strains of rabies virus differ, as has been documented in other studies [56,57], showing that the effects of different strains on autophagy could be opposite. It is likely that the discrepancy between our and Yuan’s findings [55] may be due to the different strains used, although we could not rule out other possibilities. However, for the direct antiviral activity mediated by TRIM proteins, there may be an indirect mechanism between the host and a TRIM protein. Therefore, further research is needed to identify which TRIM protein interacts with the host proteins to mediate the suppression of RABV infection.

In the “battle” between virus and host, the virus has developed a defense mechanism through interfering with host factors. Therefore, viral components may conversely target TRIM proteins to impact TRIM protein-mediated ubiquitination. For example, nucleocapsid proteins from severe acute respiratory syndrome coronavirus PRRSV (SARS-CoV) or SARS-CoV-2 interact with TRIM25 and affect TRIM25-mediated activation of RIG-I [40,43,58]. Nonstructural protein 1 from respiratory syncytial virus could interact with TRIM25 and subsequently suppress RIG-I-mediated type-I IFN signaling [41]. However, HEP-Flury proteins do not directly target TRIM25 based on our result that there are no direct interactions between HEP-Flury structural proteins and TRIM25. The expression of TRIM25 and its subsequently mediated ubiquitination are regulated by several mechanisms in virus infection. For example, long non-coding RNA XIST upregulates the expression of TRIM25 by targeting microRNA 192 in HBV infection [59]. Sendai virus infection increases the interaction between serine/threonine-protein kinase 38-like and TRIM25, which triggers RIG-I ubiquitination [60]. Ubiquitin-specific protease 15 is recruited to TRIM25 in sendai virus infection, and then deubiquitylates TRIM25 to regulate RIG-I-mediated type-I IFN response [61]. Therefore, further studies are required to uncover the host factors by which TRIM25 potentiates RIG-I-mediated type-I IFN response in HEP-Flury infection.

**Figure 7:**
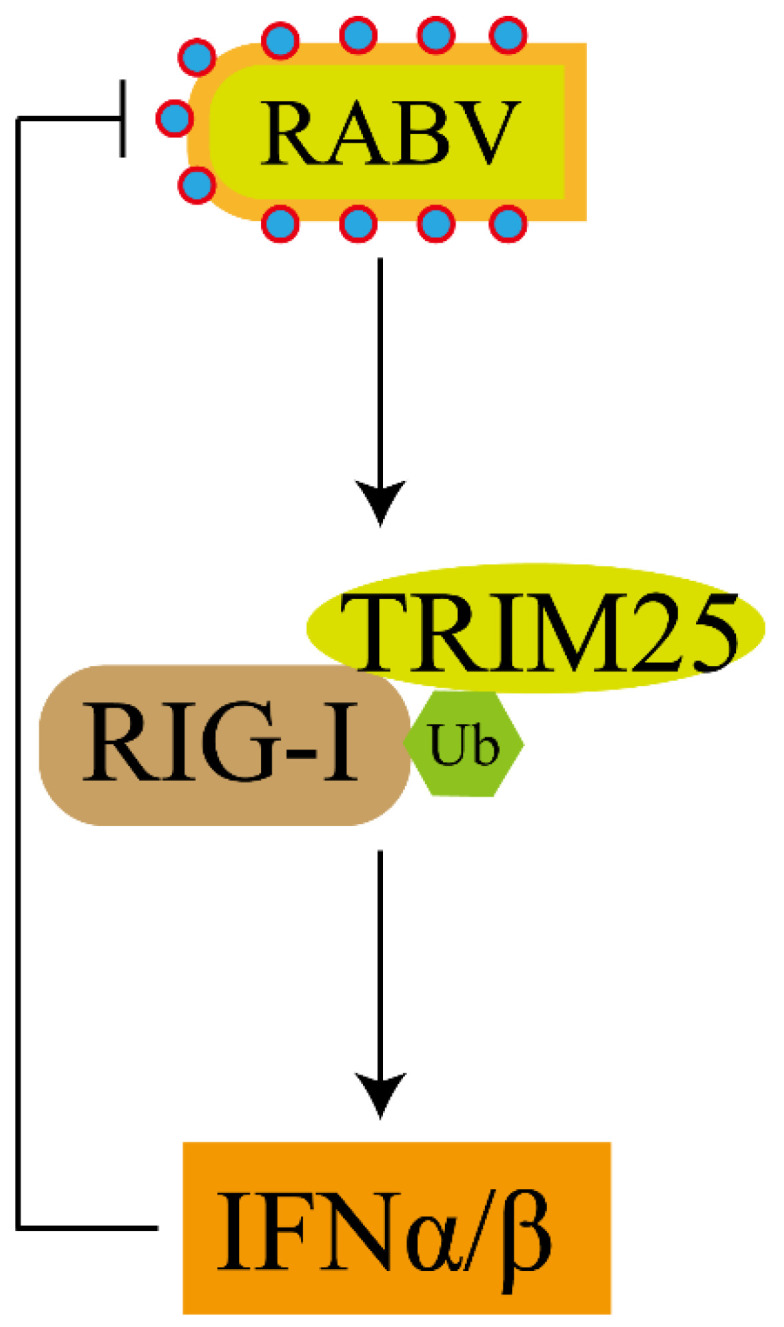
A model for the suppressive effect of TRIM25 on RABV replication by activating the RIG-I-IFN axis.

In summary, in this study, TRIM25 is identified as a novel host resistance factor for HEP-Flury infection. We demonstrated that TRIM25 inhibits HEP-Flury replication through an RIG-I-mediated type-I IFN response (Figure 7), rather than through direct interaction with HEP-Flury structural proteins. Taken together, our study provides support for the mechanism of action of TRIM25 against RABV, contributing to better understanding of the regulation mechanism of I-IFN on HEP-Flury infection. Our efforts may facilitate the development of new strategies for controlling RABV infection.

## 4. Materials and Methods

### 4.1. Viruses and Cells

RABV fixed HEP-Flury strain (gifted by Dr. He Kongwang, Jiangsu Academy of Agricultural Sciences, Nanjing, China) was propagated in N2a cells. N2a cells (Wuhan Institute of Biological Products, Wuhan, China) were cultured and passaged in RPMI 1640 medium (Gibco) containing 10% fetal bovine serum (FBS) (Gibco). HEK 293T cells were cultured and passaged in DMEM medium containing 10% FBS.

### 4.2. Antibodies, Plasmidsand siRNAs

Antibodies to Myc (sc-40) and HA (sc-7392) were purchased from Santa Cruz Biotechnology (Dallas, TX, USA); antibodies to RIG-I (#4200S) and TRIM25 (#13773S) were purchased from Cell Signaling Technology (Danvers, MA, USA); Antibody to IFNβ (ab218229) andIFNα (ab191903) was purchased from abcam (Cambridge, UK); anti-Flag antibody (AF5051), normal mouse IgG (A7028) and anti-β-actin antibody (AA128) were purchased from Beyond Technology (Shanghai, China). Anti-HEP-Flury P antibody was from our laboratory. FITC-labeled anti-RABV nucleoprotein (N) antibody was from Fujirabio Diagnostics (Malvern, PA, USA).

The pCAGGS plasmid carrying the HA tag (pCAGGS-HA), the pCAGGS plasmid carrying the Flag tag (pCAGGS-Flag) and the ubiquitin plasmid carrying the Myc tag (pcDNA3.1-Myc-Ub) were purchased from Wuhan Miaoling Biotechnology Co., Ltd. (Wuhan, China). Cloning of the TRIM25 gene from N2a cells into pCAGGS-HA using primer TRIM25-F: 5′-AGCTCATCGATGGTACCCGGGCCATGGCGGAGCTGAATCCTCT-3′ and TRIM25-R: 5′-ATTAAGATCTGCTAGCTCGAGCTATTTGGAGCAGATAGAGAGGGTG-3′ for the RIG-I gene of N2a cells was amplified using primers and cloned into the pCAGGS-Flag plasmid:RIG-I-F: 5′-GATGACGACGATAAGGAATTCATGACAGCGGAGCAGCG-3′ and RIG-I-R: 5′-ATTAAGATCTGCTAGCTCGAGTCATACGGACATTTCTGCAGGATC-3′ for overexpression of RIG-I. The structural protein gene of HEP-Flury was amplified with the primers in Table 1, and cloned into the pCAGGS-Flag vector to construct overexpression plasmid with Flag marker. The sequences of protein-targeting siRNAs and non-targeting control (NC) siRNAs used in this study are listed in Table 2 and were synthesized by Sangon Biotechnology (Shanghai, China).

### 4.3. HEP-Flury Titration

Consistent with our previous method, the titer of HEP-Flury was detected by direct immunofluorescence [62]. Briefly, 10-fold serial dilutions of HEP-Flury-infected cell supernatants were performed using RPMI 1640 medium. The diluted virus solution was inoculated into N2a cells in a 96-well plate. Cells were incubated at 37 °C for 48 h. Then, discard the supernatant and add 80% acetone to fix at −20 °C for 30 min. After the fixation, acetone was discarded and washed, and then incubated with FITC-labeled antibody against RABV N protein at 37 °C for 1 h. The cells in the 96-well plate were observed with a fluorescence microscope (AMG, Washington, DC, USA), and the titer of the virus was calculated according to the Kappa method.

### 4.4. Immunofluorescence Staining

Add 80% acetone to N2a cells, and then fix at −20 °C for 20 min. After the fixation, discard the liquid and wash three times, then add FITC-labeled anti-RABV N protein antibody, and incubate at 37 °C for 1 h. Cells were then observed and photographed using a fluorescence microscope.

### 4.5. siRNA and Plasmid DNA Transfection

Use LIPO3000 (Invitrogen, Carlsbad, CA, USA) to transfect siRNA or recombinant plasmids into HEK 293T cells or N2a cells, and use NC and blank plasmids as controls. If multiple siRNAs or plasmids need to be transfected, perform the next round of transfection 12 h after the previous siRNA or plasmid transfection.

### 4.6. Co-Immunoprecipitation

The operation was performed according to the operating instructions of the Pierce™ Cross-Linked Magnetic IP/CO-IP Kit (Thermo Fisher Science Company, Woltham, MA, USA). After the cells were transfected with the corresponding plasmid for 36 h, the cells were collected, and the cell lysate was prepared. The corresponding antibody was coupled to the cross-linked magnetic beads, and then the plasma cell lysate was incubated with the cross-linked magnetic beads at 4 °C for 10 h, and the magnetic beads coupled with normal IGg were used as a control. The protein was eluted from the magnetic beads and detected by Western blot (WB) using the corresponding antibody.

### 4.7. Western Blotting

The operation of Western blotting was consistent with that described in our past studies [63]. Briefly, IP products or cell lysates were subjected to protein separation by 12% sodium dodecyl sulfate-polyacrylamide gel electrophoresis, and proteins were transferred to polyvinylidene fluoride membranes (Millipore, Bedford, MA, USA), and the membranes were incubated with corresponding antibodies as needed. The corresponding proteins were then imaged using the Fine-do X6 chemiluminescence imaging system (Tanon, Shanghai, China).

### 4.8. Quantitative Real-Time PCR Analysis

The cells were collected, and then the total RNA in the cells was extracted using TRIzol reagent (Magen, Guangzhou, China). Total RNA was subjected to reverse transcription (RT) using Transcription First Strand cDNA Synthesis Kit (Vazyme Biotech, Nanjing, China). Real-time quantitative PCR was then carried out in a CFX connect real-time system (Bio-Rad, Hercules, CA, USA) according to the operating instructions of SYBR Green Master Mix (Vazyme Biotech). According to the transcription level of glyceraldehyde-3-phosphate dehydrogenase (GAPDH) gene, the gene to be detected was standardized and calibrated. Primer sequences for TRIM25 were used: forward, 5′-TCTGTAGGAGTCAAGGCTAAGGTG-3′, reverse, 5′-GTTGTGGGCGGTATTGTAGTCG-3′. The primer sequences for gRNA, GAPDH, IFNα, and IFNβ have been introduced in previous studies [64].

### 4.9. Statistical Analysis

Experimental data were summarized and analyzed using GraphPad Prism 6 software (GraphPad Software, San Diego, CA, USA). Student’s *t*-test was used to analyze the experimental results. *p* < 0.05 was considered statistically significant.

## Figures and Tables

**Table 1:** Primers used for the construction of Flag-tagged overexpression plasmids for HEP-Flury structural proteins.

ID	Sequence (5′-3′)
HEP-N-FLAG-F	TTCGAGCTCATCGATGGTACCATGGATGCCGACAAGAT
HEP-N-FLAG-R	AATTAATTAAGATCTGCTAGCTTATGAGTCACTCGAATACG
HEP-P-FLAG-F	TTCGAGCTCATCGATGGTACCATGAGCAAGATCTTTGTTAATCCGAGTG
HEP-P-FLAG-R	AATTAATTAAGATCTGCTAGCTTAGCATGATGTGTAGCGATCCAAG
HEP-M-FLAG-F	TTCGAGCTCATCGATGGTACCATGAACTTTCTATGTAAGATAGTGAA
HEP-M-FLAG-R	AATTAATTAAGATCTGCTAGCTTATTCTAAAAGCAGAGAAGAGTC
HEP-G1-FLAG-F	TTCGAGCTCATCGATGGTACCATGGTTCCTCAGGTTCTTTTG
HEP-G1-FLAG-R	AATTAATTAAGATCTGCTAGCTCACTTCCCCCATTTCGG
HEP-G2-FLAG-F	TTCGAGCTCATCGATGGTACCTATGTATTGATGATTGCAGG
HEP-G2-FLAG-R	AATTAATTAAGATCTGCTAGCTCACAGTCTGGTCTCG
HEP-L-FLAG-F	TTCGAGCTCATCGATGGTACCATGCTGGATCCGGGAGAG
HEP-L-FLAG-R	AATTAATTAAGATCTGCTAGCTTACAAACAACTGTAGTCTAGTAGGGA

**Table 2:** The siRNAs used in this study.

siRNA	Sense (5′-3′)	Antisense (5′-3′)
TRIM25-1	GCAAAUGUACCCAGCACAATT	UUGUGCUGGGUACAUUUGCTT
TRIM25-2	CCCACUUCUCACCUAACAATT	UUGUUAGGUGAGAAGUGGGTT
TRIM25-3	CCUCUCUAUCUGCUCCAAATT	UUUGGAGCAGAUAGAGAGGTT
RIG-I-1	CCAGAUGAUAGAUACUACAAUTT	AUUGUAGUAUCUAUCAUCUGGTT
RIG-I-2	CCUGGGUAAUGAAAGCCGAUUTT	AAUCGGCUUUCAUUACCCAGGTT
RIG-I-3	GCUGGCAUGUAUAUGUGCGUATT	UACGCACAUAUACAUGCCAGCTT
IFNα-1	GAGCCAGAUUAUCUCUUUCUATT	UAGAAAGAGAUAAUCUGGCUCTT
IFNα-2	CGUCAUUGAAUCACACCUGAUTT	AUCAGGUGUGAUUCAAUGACGTT
IFNα-3	CAGUCAUUGAAAGCCUAGAAATT	UUUCUAGGCUUUCAAUGACUGTT
IFNβ-1	GCAGAAGAGUUACACUGCCUUTT	AAGGCAGUGUAACUCUUCUGCTT
IFNβ-2	AGCCCUCUCCAUCAACUAUAATT	UUAUAGUUGAUGGAGAGGGCUTT
IFNβ-3	GCUCUCCACUUGAAGAGCUAUTT	AUAGCUCUUCAAGUGGAGAGCTT
NC	UUCUCCGAACGUGUCACGUTT	ACGUGACACGUUCGGAGAATT

## Data Availability

The data presented in this study are available on request from the corresponding author. The data are not publicly available due to their containing information that could compromise the privacy of research participants.
